# The Association Between Prolonged Use of Oral Corticosteroids and Mental Disorders: Do Steroids Have a Role in Developing Mental Disorders?

**DOI:** 10.7759/cureus.37627

**Published:** 2023-04-15

**Authors:** Mouath A Alturaymi, Omar F Almadhi, Yazeed S Alageel, Majed Bin Dayel, Mohammed S Alsubayyil, Badr F Alkhateeb

**Affiliations:** 1 College of Medicine, King Saud Bin Abdulaziz University for Health Sciences, Riyadh, SAU

**Keywords:** psychological sexual dysfunction, anxiety, depression, mental disorder, corticosteroid

## Abstract

Background

The use of oral corticosteroids has been linked to a variety of mental health problems, including mental disorders such as anxiety, depression, and psychosis. In a recent study, researchers investigated the prevalence of steroid-induced neuropsychiatric side effects in a population of patients receiving steroid treatment. This study aimed to evaluate the association between steroids and mental disorders among patients in King Abdulaziz Medical City.

Methods

A retrospective descriptive study was conducted in King Abdulaziz Medical City, Riyadh, Saudi Arabia from January 2016 to November 2022. Data were acquired from all the registered inpatients and outpatients who were using oral corticosteroids for more than 28 days. Data were entered into the Statistical Package for the Social Sciences (SPSS) version 23 (IBM Corp, Armonk, NY) for analysis after data collection. The numerical data were presented as mean and standard deviation and a test of significance was applied (p<0.05). For categorical data, frequency and percentages were computed. The chi-square test of significance was applied across groups and the test of significance was computed (p<0.05).

Results

The study included 3138 patients who were using oral corticosteroids for more than 28 days, and electronic medical records were screened for the presence of a concurrent mental disorder. Moreover, 142 out of 3138 developed a mental disorder after the prolonged use of oral corticosteroids. The most commonly reported mental disorder was anxiety followed by psychological sexual dysfunction and depressive disorders. Gender, age, and type of steroid prescribed had a significant association (p<0.001) with the development of psychiatric adverse events.

Conclusion

These findings highlight the importance of monitoring patients who are receiving oral corticosteroid treatment for signs of mental health problems and adjusting treatment as needed. Healthcare providers should also educate patients about the potential risks associated with corticosteroids and encourage them to seek medical attention if they experience any mental health symptoms.

## Introduction

Steroids' therapeutic roles are linked to their powerful anti-inflammatory and immune-modulating characteristics. The side effects of steroids are prevalent and problematic, ranging from a mild case of acne to Cushing syndrome, which can lead to diabetes mellitus and potentially life-threatening cardiac disease if left untreated [1]. Steroids are widely used to treat a variety of inflammatory and autoimmune diseases, including rheumatic diseases such as rheumatoid arthritis (RA) and systemic lupus erythematosus, inflammatory diseases of the upper airways (rhinitis, chronic rhinosinusitis), and pulmonary inflammatory diseases such as bronchial asthma and chronic obstructive pulmonary disease [2]. Psychiatric adverse effects of corticosteroid treatment include depression, anxiety, delirium, panic disorder, and many other psychiatric problems. Although psychotropic medication may be required to manage these symptoms, the prognosis improves once the corticosteroids are lowered or eliminated. Adverse effects occur in up to 90% of patients who have been taking corticosteroids for more than 60 days [3]. In one study, manic symptoms were not consistent among people, with most demonstrating no psychological change while a few showed significant impacts [4]. It has been documented that systemic corticosteroid administration causes cognitive impairment that might progress into dementia or delirium [5]. Corticosteroids have considerable side effects; in particular, delirium, depression, mania, psychosis, and cognitive/memory impairment might develop [6]. Corticosteroid-induced mental disorders, which can cause significant behavioral and emotional issues, are undesirable side effects of corticosteroids [7]. Although steroids are highly efficient, they have numerous physical and psychological side effects. A study conducted in Saudi Arabia described sort of steroid use and its effects on mental health or mental disorder [8].

The reported incidence of mental problems following steroid usage ranges from 2% to 60% [9]. A study that included 463 patients who were using steroid therapy showed that the prevalence of psychiatric reactions due to the use of prednisone is 1.3% [10]. In a study of lupus nephritis patients, 32% of those given prednisolone experienced severe psychosis, but only 3.8% of those who were not given prednisolone acquired severe psychosis [11]. Moreover, a study that included 93 patients receiving oral corticosteroids showed that 67 patients suffered from insomnia, 25 patients suffered from delirium, and 15 patients suffered from depression after steroids were prescribed to them [12]. According to placebo-controlled trials, one-third of individuals using glucocorticoids have substantial mood and sleep disturbances [13]. More crucially, up to 20% of patients on high doses of glucocorticoids experience mental illnesses such as depression, mania, psychosis, or a mixed affective state [13]. A double-blind placebo-controlled experiment of corticosteroid treatment in healthy persons found that 75% of patients experienced mood and cognition problems that resolved after the steroids were withdrawn [14].

Due to the lack of studies conducted in Saudi Arabia regarding this issue, this study aimed to evaluate the association between steroids and mental disorders among patients in King Abdulaziz Medical City (KAMC), Riyadh, Saudi Arabia.

## Materials and methods

A retrospective descriptive study was conducted in King Abdulaziz Medical City, Riyadh, Saudi Arabia from January 2016 to November 2022. All the registered inpatients and outpatients in King Abdulaziz Medical City, Riyadh who were prescribed steroids were included in this study. The inclusion criteria included all patients aged 18 years and more who were using oral corticosteroids for more than 28 days. All patients who used steroids for less than one month or used other forms of corticosteroids such as inhaled corticosteroids were excluded. This study included a total of 3138 patients. The sampling technique was used as non-probability consecutive sampling. The investigators collected data using a chart review method utilizing a data collection sheet at King Abdulaziz Medical City. Data were gathered at the inpatient and outpatient departments for the purpose of assessing prescribed steroids and the development of mental disorders over a specific period. Data were entered into the Statistical Package for the Social Sciences (SPSS) version 23 (IBM Corp, Armonk, NY) for analysis after data collection. The numerical data were presented as mean and standard deviation and a test of significance was applied (p<0.05). For categorical data, frequency and percentages were computed. The chi-square test of significance was applied across groups and the test of significance was computed (p<0.05). This study was approved by King Abdullah International Medical Research Center (KAIMRC) with the approval number IRB/2812/22.

## Results

The study on hands included an overall of 3138 patients who were using oral corticosteroids for more than four weeks, of which 1082 (34.5%) were males and 2056 (65.5%) were females. The mean age of the included steroid-user patients was 47.06±17.093. Most of our patients were aged from 38 to 57 years, followed by 18-37 years, respectively.

The overall prevalence of mental disorders among patients using steroids was 5.5% (n=142). The mean age of the patients who developed a mental disorder during or after the period of steroid use was 49.56±17.705. As shown in Table 1, most of them are females, 89 (62.67%), vs 53 (37.32%) males. The mean dose of steroids among those with a mental disorder was 7.05±6.627 mg.

**Table 1:** Mental disorders and distribution of gender. ADHD, attention-deficit/hyperactivity disorder; PTSD, post-traumatic stress disorder.

		Gender		Total
		Male	Female	
Mental disorder	Depressive disorder	7	17	24
	Cognitive disorder	2	6	8
	Psychosis	0	2	2
	Mixed anxiety and depressive disorder	2	10	12
	Somatization	0	1	1
	Panic disorder	0	3	3
	ADHD	0	2	2
	Adjustment disorders	0	1	1
	Nonorganic insomnia	1	1	2
	Globus pharyngeus	0	1	1
	Sleep bruxism	0	2	2
	Depressive episode	6	11	17
	Trichotillomania	0	1	1
	Conduct disorder	1	1	2
	Non-organic enuresis	1	0	1
	Unspecified mental disorder	0	2	2
	Anxiety disorder	6	24	30
	PTSD	2	1	3
	Schizophrenia	1	1	2
	Psychological sexual dysfunction	24	0	24
	Dysthymia	0	2	2
Total		53	89	142

Table 2 shows that the most commonly encountered mental disorder among the corticosteroid users was anxiety disorder with a prevalence of 0.95%, followed by sexual dysfunction (0.76%) and then depressive disorder (0.7%). The mean duration of oral corticosteroid use was 93.01±54.91 days, with most of the patients using oral corticosteroids for a duration of 28-90 days, respectively.

**Table 2:** Prevalence of mental disorder after steroid therapy ADHD, attention-deficit/hyperactivity disorder; PTSD, post-traumatic stress disorder.

Mental disorder	Number of patients	Prevalence (%)
Depressive disorder	22	0.7
Cognitive disorder	8	0.25
Psychosis	2	0.06
Mixed anxiety and depressive disorder	12	0.38
Somatization	1	0.03
Panic disorder	3	0.09
ADHD	2	0.06
Adjustment disorders	1	0.03
Non-organic insomnia	2	0.06
Globus pharynges	1	0.03
Sleep bruxism	2	0.06
Depressive episode	17	0.54
Trichotillomania	1	0.03
Conduct disorder	2	0.06
Non-organic enuresis	1	0.03
Unspecified mental disorder	2	0.06
Anxiety disorder	30	0.95
PTSD	3	0.09
Schizophrenia	2	0.06
Psychological sexual dysfunction	24	0.76
Dysthymia	4	0.12

Moreover, prednisolone was the most common steroid used with a percentage of 81.5%, followed by hydrocortisone (9%) and dexamethasone (7%).

As shown in Table 3, we found no statistical association between the type of mental disorder and age, as the p-value was 0.751. In contrast, there was a statistical association between types of mental disorders and gender as females were more prone to develop anxiety disorders after using steroids (p=0.003).

**Table 3:** Association between mental disorders, age, and gender. ADHD, attention-deficit/hyperactivity disorder; PTSD, post-traumatic stress disorder.

Disorder	Age					Gender	
	18-37 years	38-57 years	58-77 years	78-97 years	More than 97 years	Male	Female
Depressive disorder	3	11	7	1	0	6	16
Cognitive disorder	0	4	4	0	0	2	6
Psychosis	1	0	1	0	0	0	2
Mixed anxiety and depressive	7	4	1	0	0	2	10
Somatization	0	0	1	0	0	0	1
Panic disorder	2	1	0	0	0	0	3
ADHD	1	0	1	0	0	0	2
Adjustment disorders	0	1	0	0	0	0	1
Non-organic insomnia	0	1	1	0	0	1	1
Globus pharynges	1	0	0	0	0	0	1
Sleep bruxism	0	2	0	0	0	0	2
Depressive episode	3	6	7	1	0	6	11
Trichotillomania	1	0	0	0	0	0	1
Conduct disorder	1	0	0	1	0	1	1
Non-organic enuresis	1	0	0	0	0	1	0
Unspecified mental disorder	2	0	0	0	0	0	2
Anxiety disorder	9	8	11	2	0	6	24
PTSD	1	1	0	1	0	2	1
Schizophrenia	1	0	1	0	0	1	1
Sexual dysfunction	7	9	8	0	0	24	0
Dysthymia	0	4	0	0	0	0	4
p-Value	0.751						0.003

Also, Table 4 illustrates that there was no statistical association between the occurrence of mental disorders and specific types of steroids (p=0.689).

**Table 4:** Type of steroid use in different mental disorders. ADHD, attention-deficit/hyperactivity disorder; PTSD, post-traumatic stress disorder.

Mental disorder	Type of steroid prescribed						p-Value
	Betamethasone	Budesonide	Dexamethasone	Hydrocortisone	Methylprednisolone	Prednisolone	
Depressive disorder	0	0	1	1	0	20	0.689
Cognitive disorder	0	0	0	0	0	8	
Psychosis	0	0	1	0	0	1	
Mixed anxiety and depressive disorder	0	1	0	0	0	11	
Somatization	0	0	0	0	0	1	
Panic disorder	0	0	1	0	0	2	
ADHD	0	0	1	0	0	1	
Adjustment disorders	0	0	0	0	0	1	
Non-organic insomnia	0	0	0	0	0	2	
Globus pharynges	0	0	0	0	0	1	
Sleep bruxism	0	0	0	0	0	2	
Depressive episode	0	0	1	2	0	14	
Trichotillomania	0	0	0	0	0	1	
Conduct disorder	0	0	0	0	0	2	
Non-organic enuresis	0	0	1	0	0	0	
Unspecified mental disorder	0	0	0	0	0	2	
Anxiety disorder	0	0	2	2	0	26	
PTSD	0	0	1	0	0	2	
Schizophrenia	0	0	1	0	0	1	
Sexual dysfunction	0	0	0	3	0	21	
Dysthymia	0	0	0	0	0	4	

## Discussion

The current study aimed to assess the prevalence of mental disorders after the prolonged use of oral steroids and to evaluate the association between long-term steroid use and mental disorders. The findings of this study showed that after prescription the effect of steroids was associated with various mental disorders. The most prevalent type of psychiatric disorder in this study was anxiety disorder (0.95%) followed by psychological sexual dysfunction (0.76%) and depressive episodes (0.70%). The most common corticosteroid that was associated with the development of a psychiatric disorder is prednisolone. Female gender showed a statistically significant association with the occurrence of psychiatric disorders in general, as females were more prone to acquire psychiatric adverse effects when using corticosteroids.

The fifth edition of the Diagnostic and Statistical Manual of Mental Disorders classifies steroid-induced mental disorders as a type of substance/medication-induced psychotic disorder [15]. According to one study, before beginning corticosteroid medication, a history and physical examination are required to examine any risk factors or preexisting illnesses that could be exacerbated by corticosteroid treatment [16]. Diabetes, hypertension, congestive heart failure, hyperlipidemia, mental issues, and osteoporosis are all included in this workup [16].

Previous works of literature reporting the prevalence of steroid-induced psychiatric adverse effects have cited ranges between 1.8% and 57% [17]. One meta-analysis showed that the average incidence of severe psychiatric side effects of steroids is 5.7%, which is clearly in line with the findings of our study as we illustrated that the prevalence of steroid-induced psychiatric illness is 5.5% [18].

A meta-analysis of randomized controlled trials has provided firm confirmation that they can cause psychosis if used for a prolonged period [19]. In our study, psychosis was not one of the common side effects of steroids as only two patients out of 3138 steroid users developed psychosis after the start of steroid therapy. It was interesting to show in the current study that most males had psychological sexual dysfunctions. Psychological sexual dysfunction could be regarded as any psychological issue that hinders you or your partner from experiencing sexual fulfillment. Male sexual dysfunction is a prevalent health issue that affects men of all ages, although it becomes more common as they become older. The findings of the current study were in line with another study's findings that sexual dysfunction is apparently more common in persons with mental problems than in the normal population, with rates ranging from 40% to 96% [20-23].

The current study showed that the majority of patients who developed a mental disorder were using prednisolone. The findings were similar to that of another study that found that prednisolone medication causes symptoms of hypomania, mania, depression, and psychosis, as well as cognitive impairments, specifically deficiencies in verbal or declarative memory [24]. Psychiatric symptoms tend to be dose-dependent and often manifest themselves during the first few weeks of therapy [24]. The findings of this study were consistent with another study that showed that prednisone, a prodrug of prednisolone, has been linked to neuropsychiatric symptoms such as depression, mania, agitation, delirium, dementia, psychosis, and a variety of other affective, behavioral, and cognitive abnormalities. Although the evidence suggests that patients using 40 mg or more of prednisone per day are at a higher risk for steroid-induced psychosis, people taking 40 mg are nonetheless at risk, and steroid-induced psychosis should not be ruled out [25]. Another study suggested that symptoms such as euphoria, sleeplessness, mood swings, personality changes, severe depression, and psychosis, known as corticosteroid-induced psychosis, are thought to occur in 5%-18% of individuals treated with corticosteroids [17].

A control-based study evaluated the effects of corticosteroid therapy on mood changes; they highlighted that 60% of the corticosteroid-receiving group met the DSM5 diagnostic criteria for corticosteroid-induced mood disorder [26]. Moreover, a study included 550 patients receiving prednisolone and 449 control group, and the ophthalmological patients demonstrated higher rates of mental disturbance as 26% developed mania and 10% depression; however, no psychotic features were observed during steroid therapy [27]. These findings are in line with ours, as most patients who developed psychiatric adverse effects of corticosteroid therapy acquired mood changes, depression, and anxiety respectively.

This study has some limitations, one of which is the electronic charts being the only source for the data gathering. Also, this study is conducted in a single center, which is another limitation. Thus, we recommend conducting this study on a larger sample size including more tertiary centers from different cities to obtain the best view.

## Conclusions

Corticosteroids are used to treat a range of common ailments. As a result, all physicians are concerned about the psychological adverse effects of these drugs. The statistics on incidence and prevalence are limited, and few controlled studies evaluating treatment for steroid-induced psychiatric issues have been conducted. The available data, however, indicate that psychiatric symptoms during corticosteroid therapy are dosage dependent, frequently arise early in treatment, and include mania, depression, lability, and psychosis. Further study on the use of corticosteroids to treat both mood and cognitive problems is required. Steroid-associated mental disorders are a well-documented side effect of long-term steroid use, particularly at high doses. These disorders can include anxiety, depression, and psychosis. The prevalence of these disorders can vary depending on the patient population, the type and dose of steroids used, and other factors. It is important for healthcare providers to monitor patients who are taking steroids for signs of mental health problems and to adjust treatment as needed. Patients who experience mental health symptoms while taking steroids should seek medical attention.
